# Editorial: Integrative Brain Function Down Under

**DOI:** 10.3389/fncir.2020.00048

**Published:** 2020-08-14

**Authors:** Pankaj Sah, Greg J. Stuart, Gary F. Egan

**Affiliations:** ^1^Queensland Brain Institute, The University of Queensland, Brisbane, QLD, Australia; ^2^John Curtin School of Medical Research, Australian National University, Canberra, ACT, Australia; ^3^Monash Biomedical Imaging and School of Psychological Sciences, Monash University, Melbourne, VIC, Australia

**Keywords:** brain structure and function, neural networks, ion channel activation, prefrontal cortex, brain systems, functional encoding brain networks

Despite decades of research, how the brain interacts with the world is still one of the greatest challenges of the twenty-first century. To address this challenge, Australia's leading brain researchers are investigating brain structure and function at multiple scales, from single cells and synapses, to circuits and networks, to whole brain systems.

In this Research Topic, we are pleased to present a collection of articles and reviews from Australian neuroscientists, engineers, and computer scientists, covering a multitude of topics ranging from ion channel function in single neurons to sensory information processing within neural networks. The aim of this research is to increase our understanding of how the brain integrates information across multiple levels. Key to this process is the development of new experimental instruments and computational tools. Hopefully, the new insights obtained will also aid development of approaches to repair and restore function to the damaged brain.

In order to understand integrative brain function, it is critical to understand how the brain processes information. Information processing in the brain relies on spiking activity in single neurons, which requires the movement of charged ions through ion channels in neuronal membranes. Autuori et al. investigate ion flow through small conductance calcium-activated potassium channels (so-called SK channels), which contribute to the after hyperpolarization that follows spike activity in many neuronal cell types. The team identified that the rSK1 protein acts as a chaperone for rSK2 channels, indicating that expression of the rSK1 gene may control the level of functional SK current in neurons. To further gain insight into information processing in the brain, an understanding of how populations of neurons encode information in their patterns of spiking activity is essential. Triplett and Goodhill review recent methods for extracting variables that quantitatively describe how sensory information is encoded. In particular, they discuss methods for estimating receptive fields, modeling neural population dynamics and inferring low dimensional latent structure from neuronal populations. In a related study, Zavitz et al. present an overview of some of the most promising analytical approaches for making inferences from population recordings in multiple brain areas, such as dimensionality reduction and changes in correlated variability. Hadjidimitrakis et al. review the evidence related to functional communication between subregions of the posterior parietal cortex and how recordings in this region can be used to decode movement goals. These data suggest that the posterior parietal cortex works as a dynamic network of sensorimotor loci that combine multiple signals which work in concert to guide motor behavior, and raises the possibility of using parietal neuron activity to better drive neuroprosthetic devices for motor control.

The brain integrates and processes a massive amount of sensory information to guide behavior crucial for survival. Oestreich et al. investigate the connections between brain regions activated by speech. They identified that structurally connected brain regions are also functionally engaged by externally-generated and temporally-predictable speech patterns. The research provides evidence that the brain continually predicts incoming sensory events based on past experience in order to respond to unexpected events in a fast and efficient manner. Work by Lian et al. explores how the visual system codes visual stimuli. Using a biologically plausible model they show how complex experimental phenomena, such as the shape of receptive fields and contrast invariance of orientation tuning, can be implemented in primary visual cortex by sparse coding. In a related article, Chaplin et al. compare the representations of space and motion in the visual and auditory cortex, and examine how single neurons in these two areas encode the direction of motion. They discuss how humans integrate audio and visual motion cues, and the regions of the cortex that may mediate this process.

Several articles in this collection are dedicated to developing new approaches and building better models of integrative brain function. For example, Vidyasagar et al. propose a model of how the claustrum orchestrates and integrates activity across different cortical areas by boosting synchronized oscillations between these areas. Gollo et al. present a non-linear hierarchical model that provides unique insights into the brain architecture underlying the representation and appraisal of perceptual belief and precision in the prefrontal cortex. Jacques et al. describe a novel approach to precisely map molecular markers in neuronal networks through quantitative topographic measurement. This approach can be used to gain a greater understanding of functional encoding within sub-nuclei during memory formation and may prove advantageous for studying the cellular basis of addiction as well as pathological memory models. Finally, Arnatkevičiute et al. review studies investigating gene expression patterns associated with hub connectivity in neural networks and present evidence that some of these expression patterns are conserved across species and scales. Together, these studies provide new models of brain networks which aid our understanding of how the brain integrates information across multiple brain regions.

The articles in this Research Topic study the relationship between brain activity and behavior at multiple spatial and temporal scales—from single cell electrical and biochemical activity to patterns of activity in large scale circuits and networks. In doing so they help to build an integrated model of how the brain processes information and thereby contribute to a deeper understanding of how the brain interacts with the world.

## Author Contributions

PS initiated the Editorial draft, to which both GS and GE contributed and edited. All authors contributed to the article and approved the submitted version.

## Conflict of Interest

The authors declare that the research was conducted in the absence of any commercial or financial relationships that could be construed as a potential conflict of interest.

